# Osimertinib Optimal Dosing Through Population Pharmacokinetics in Virtual Patients with NSCLC: An Approach Integrating Multiple Studies

**DOI:** 10.3390/pharmaceutics18101332

**Published:** 2026-10-20

**Authors:** Ji Woo Lim, Heungjo Kim, Hongjae Lee, Hyeeun Kim, Kyubin Kim, Kyongkuk Ryu, Min Jung Chang

**Affiliations:** 1 Department of Integrative Biotechnology, College of Pharmacy, Yonsei University, Incheon 21983, Republic of Korea; lmjw@yonsei.ac.kr; 2 Department of Pharmacy and Yonsei Institute of Pharmaceutical Sciences, Yonsei University, Incheon 21983, Republic of Korea; 3 Department of Pharmaceutical Medicine and Regulatory Sciences, College of Pharmacy, Yonsei University, Incheon 21983, Republic of Korea; 4 Graduate Program of Industrial Pharmaceutical Science, College of Pharmacy, Yonsei University, Incheon 21983, Republic of Korea

**Keywords:** osimertinib, pharmacometrics, population pharmacokinetics, integrated popPK, NSCLC, dose optimization

## Abstract

**Background/Objectives:** Osimertinib is a third-generation tyrosine kinase inhibitor targeting epidermal growth factor receptor (EGFR) T790M mutations in metastatic non-small cell lung cancer (NSCLC). A comprehensive population pharmacokinetic (popPK) model may inform dosing across patient populations. The objective was to develop an integrated popPK model for osimertinib in virtual patients with NSCLC and to evaluate the influence of patient characteristics on PK variability. **Methods:** Data were extracted from four published popPK studies of osimertinib in patients with NSCLC. A virtual cohort of 1000 patients from each study was simulated using the corresponding final popPK model, with covariate distributions reconstructed from the reported demographics, resulting in a total virtual cohort of 4000 patients. The primary outcome was the development of a popPK model using nonlinear mixed-effects modeling. Demographic and clinical covariates were evaluated for effects on PK variability. **Results:** A parent-metabolite one-compartment model best described osimertinib popPK. Body weight and serum albumin were significant covariates, with lower body weight and lower albumin concentrations associated with increased osimertinib plasma exposure. However, simulations demonstrated that the standard 80 mg once-daily dose maintained plasma concentrations within the literature-informed reference interval across all body weight and albumin subgroups. **Conclusions:** This popPK model suggests that despite significant effects of body weight and albumin on osimertinib exposure, routine dose adjustment may not be required. The standard 80 mg once-daily regimen maintains concentrations within the reference interval across diverse virtual patient populations with NSCLC, supporting current dosing recommendations. These model-informed findings warrant prospective validation with real-world clinical data to confirm generalizability.

## 1. Introduction

Non-small-cell lung cancer (NSCLC) is a leading cause of lung cancer-related deaths worldwide, accounting for approximately 80–85% of all cases. The World Health Organization (WHO) identifies lung cancer as the leading cause of cancer-related mortality globally, with NSCLC being the predominant type [1]. Despite advancements in diagnosis and treatment, the prognosis for patients with advanced NSCLC remains poor, underscoring the need for further research and enhanced therapeutic strategies. The high prevalence and poor prognosis of NSCLC substantially contribute to the global lung cancer mortality rate [2]. Various targeted therapies and immunotherapies have been explored for NSCLC, including treatments tailored to genetic and molecular characteristics [2,3]. The National Comprehensive Cancer Network (NCCN) recommends an initial evaluation that includes biomarker testing to assess the presence of programmed death-ligand 1 (PD-L1), epidermal growth factor receptor (EGFR) mutations, and anaplastic lymphoma kinase (ALK) rearrangements. Targeted therapies, such as osimertinib, are recommended for treatment [4]. Depending on resistance levels or disease progression, alternative options, including additional targeted therapies, immunotherapy, and participation in clinical trials, may be considered. Typically, docetaxel is administered as a secondary treatment following platinum-based doublet chemotherapy [5].

Osimertinib, a third-generation EGFR tyrosine kinase inhibitor (TKI), was developed to target the T790M mutation, which is commonly associated with resistance to first-line treatments. In patients with disease progression despite or after first-generation TKI treatment, osimertinib has shown substantial efficacy, extending progression-free survival while maintaining a favorable safety profile. As a third-generation EGFR inhibitor, osimertinib offers high response rates and longer survival for patients with EGFR mutations. Effective monitoring of patient responses and pharmacokinetic data is crucial to ensure optimal therapeutic outcomes and minimize adverse effects. The drug was designed to selectively inhibit both EGFR mutations that drive tumor growth and secondary T790M mutations that cause resistance with high precision [6,7].

The recommended dose of osimertinib is 80 mg/day, a regimen optimized in several clinical trials to balance efficacy and tolerability. Maintaining the recommended dose is crucial to minimize the risk of adverse effects, such as rash or diarrhea, commonly associated with EGFR inhibitors, while ensuring effective plasma levels for sustained inhibition of the mutant EGFR receptor [8,9,10].

Osimertinib exhibits extensive penetration into lung tissue, which is essential for effectively targeting tumor cells in NSCLC. The drug is primarily metabolized in the liver by CYP3A4 to form two active metabolites, AZ5104 and AZ7550, which together account for approximately 10% of the osimertinib dose [11]. Despite the pharmacological activities of these metabolites, their levels in the body are relatively low, suggesting they play a secondary role in drug efficacy [12]. Osimertinib absorption is slow, with exposure increasing proportionally with doses between 20 and 240 mg. The drug is extensively distributed and has low-to-moderate clearance and an average half-life of 48.3 h. Steady-state concentrations are achieved within 15 days of dosing, consistent with single-dose pharmacokinetics. The peak-to-trough ratio of osimertinib is 1.6 [13].

A prospective cohort study reported that severe toxicity risk significantly increased when the minimum steady-state plasma concentration of osimertinib exceeded 259 ng/mL. Major side effects include rash, diarrhea, and prolongation of the cardiac QTc interval. Reducing the dose to 40 mg can decrease the risk of severe toxicity by up to 53%. However, due to the long half-life of osimertinib, plasma levels may remain above the toxicity threshold for an extended period following dose adjustment. Previous analyses have not systematically quantified whether patient characteristics, alone or in combination, can bring steady-state concentrations above the toxicity threshold [14].

In this study, we aim to describe the integrated population pharmacokinetic profile of osimertinib in patients with NSCLC using a virtual patient cohort and to evaluate the relevance of the developed pharmacokinetic model for patient characteristics and clinical status. Additionally, model simulations were used to evaluate whether the approved 80 mg dose maintains steady-state trough concentrations within a literature-informed reference interval across patient characteristics.

## 2. Materials and Methods

### 2.1. Model Study Selection

A comprehensive literature review was conducted using PubMed, Embase, and Cochrane to explore various biological variables, guided by the PICO method and the keywords listed in Table S1. This review included studies with diverse patient populations and demographic characteristics that affected the pharmacokinetic model. To reduce confounding variables, studies involving the concurrent administration of drugs from the same class were excluded, focusing solely on patients with NSCLC.

### 2.2. Data Extraction

In this study, virtual patients were generated using final models derived from selected population pharmacokinetic (popPK) studies. This simulation-based approach, generating virtual patient cohorts from published popPK model parameters, has been previously applied to characterize PK variability and inform dosing strategies for EGFR-TKIs and other therapeutic agents [15,16,17]. For each final model, plasma concentration–time simulations were conducted using the mrgsolve package (version 2.0.1) in R (version 4.5.2). Each source model was simulated in its own published structure, and the resulting concentration–time profiles were combined into a single dataset for model development. Metabolite concentrations were simulated only from the two models that included a metabolite compartment. A total of 4000 virtual patients, 1000 from each study, were created, and their characteristics were summarized in Table 1.

Ordinary differential equation models were employed to generate time–concentration profiles of osimertinib for each study. With respect to dosing, a regimen of 80 mg once daily for 19 consecutive days was simulated to achieve steady-state concentrations. Only covariates reported by all four source studies were evaluated. Covariate effects in the source models for which no virtual covariate was generated (ethnicity in Brown et al. [18] and the laboratory covariates in Agema et al. [14]) were held at their reference values. Each continuous covariate was drawn independently from a normal distribution defined by the mean and standard deviation (SD) reported in the source publication. For studies reporting the median and interquartile range (IQR), the median served as the mean, with the SD approximated as IQR/1.35 [19]. Generated values were constrained to the observed ranges reported by Brown et al. [18].

**Table 1:** Characteristics of included studies.

Study	K. Brown et al. [18]	Rodier, T. et al. [20]	Agema et al. [14]	Ishikawa et al. [21]	Simulated Characteristics
	N or mean (%)	N or median (%)	N or median (%)	N or median (%)	N or mean (%)
Total number of subjects (n)	778	87	159	53	
Sex (n)					
Male	491 (63.1)	27 (31.0)	57 (36)	21 (40)	1802 (45)
Female	287 (36.9)	60 (69.0)	102 (64)	32 (60)	2198 (55)
Age (year)	60.2 (11.9 ^a^)	66.1 (61.5–70.9 ^b^)	66 (60–75 ^c^)	69 (60–74 ^c^)	64.41 (12.24)
Body weight (kg)	63.3 (14.3 ^a^)		69 (60–80 ^c^)	55 (49–64 ^c^)	64.63 (12.72)
BMI (kg/m^2^)	23.7 (4.1 ^a^)	22.7 (21.5–24.0 ^b^)			
Starting dose (mg/day)					
20	54 (6.9)	0			
40	73 (9.4)	4 (4.6)	21 *	5 *	
80	594 (76.2)	80 (92.0)	138	47	4000
120	4 (0.5)	3 (3.4)			
160	111 (14.2)	0			
240	21 (2.7)	0			
TKI treatment line					
First-line treatment		28 (32.2)	66 (41)	37 (70)	
Second-line treatment		47 (54.0)	79 (50)	16 ** (30)	
Third-line treatment		12 (13.8)	14 (9)		
Laboratory values					
Albumin (g/dL)	3.84 (0.53 ^a^)	3.84 (3.72–3.97 ^b^)	4.0 (3.7–4.3 ^c^)	4.0 (3.8–4.3 ^c^)	3.87 (0.46)
ALT (U/L)	21.8 (18.5 ^a^)	26.0 (21.0–30.0 ^b^)	21 (15–30 ^c^)	17 (12–23 ^c^)	28.99 (15.44)
AST (U/L)	26.7 (16.7 ^a^)	25.4 (23.2–28.1 ^b^)	25 (21–31 ^c^)	23 (19–28 ^c^)	30.99 (15.23)
Serum Creatinine (µmol/L)	85.0 (28.2 ^a^)	82.0 (70.0–94.0 ^b^)	84 (71–97 ^c^)	73.4 (61.9–93.7 ^c^)	84.82 (24.85)

ALT, alanine aminotransferase; AST, aspartate aminotransferase; BMI, body mass index; * dose reduction (40 mg/day or 80 mg/2 days); ** second- or later-line treatment; ^a^ SD, standard deviation; ^b^ 25th–75th percentile; ^c^ IQR, interquartile range.

The proportions for the categorical covariate sex were maintained as previously reported. Rodier et al. [20] reported body mass index (BMI) instead of body weight. Height was assigned as the mean height for each sex and age group in the National Health and Nutrition Examination Survey 2015–2018 [22]. These reference data were used because most patients in the cohort of Rodier et al. [20] were non-Asian. Patients younger than 50 or older than 79 years were assigned the nearest age group. Body weight was calculated as BMI multiplied by the square of the assigned height. Individual variation in height within each group was not represented. The compartment structure, absorption, and elimination rates were determined based on the model study.

All observations used for model development and validation were simulated rather than reconstructed from patient data. Each dependent variable value was generated as the individual prediction plus a residual error term. A common proportional residual error of 15% was applied in place of the study-specific values in Table 2. This common error specification was applied consistently across the four source models to generate the simulated observations. Concentrations were sampled every 2 h during the first and last four days of the 19-day regimen. On the intervening days, sampling was once daily.

### 2.3. Model Development and Validation

Population pharmacokinetic (PK) analyses were performed using nonlinear mixed-effects models (NONMEM 7.6.0, ICON Development Solutions) with the first-order conditional estimation method with interaction. The model predictions included parent and metabolite apparent oral clearance (CL/F and CLm/F, respectively), apparent volume of distribution (V/F and Vm/F), and absorption rate constant (Ka).

We employed a one-compartment PK model with first-order absorption and elimination linked to a one-compartment metabolite model (ADVAN6 TOL4) to describe osimertinib PK across several primary studies. The absorption rate constant was estimated from the combined dataset and then fixed. We assessed various interindividual variability (IIV) models, including additive, proportional, and combined error models, using objective function values (OFVs), goodness-of-fit (GOF) plots, and parameter standard errors. Scatter plots were used to explore relationships between individual PK parameter estimates and covariates (sex, weight, and serum albumin), with continuous covariates centered on their median values.

We developed an initial structural model and subsequently refined it. A stepwise covariate modeling (SCM) procedure was then applied, retaining covariates that decreased the objective function value by 3.84 or more in forward selection (χ^2^ test, *p* < 0.05) and that increased it by 6.63 or more upon removal in backward elimination (χ^2^ test, *p* < 0.01) [23]. Candidate covariates in the SCM were sex, age, body weight, serum albumin, alanine aminotransferase (ALT), aspartate aminotransferase (AST), and serum creatinine. Study was also tested as a categorical covariate to assess differences between the source models.

Model evaluation comprised internal validation using GOF diagnostics, visual predictive checks (VPCs), and bootstrapping with 1000 simulations, with final parameter estimates compared against bootstrap medians and 95% confidence intervals (CIs). To assess parameter stability and potential overfitting, the 4000 virtual patients were split into a model-building (MB) set (80%, *n* = 3200) and a validation (VAL) set (20%, *n* = 800), with models fit on each subset used to predict the other. Predictive accuracy and potential overfitting were quantified using root mean square error (RMSE), mean absolute error (MAE), and bias.

**Table 2:** Final popPK parameter estimates of included studies.

	K. Brown et al. [18]		Rodier, T. et al. [20]		Agema et al. [14]		Ishikawa et al. [21]	
Parameter	Estimate	%RSE	Estimate	%RSE	Estimate	%RSE	Estimate	SE
k_a_ (h^−1^)	0.24	4.9	0.24 (fixed)	-	0.332	30.7	0.334	0.1
CL/F (L/h)	14.2	1.8	13.7	7.2	14.50	2.4	17.37	0.886
V/F (L)	986	2.8	974	17.5	1150	7.4	1271	323.4
CLm/F (L/h)	31.5	2.7	-	-	-	-	44.41	2.323
Vm/F (L)	207	4.4	-	-	-	-	589.0	23.61
Covariate effect on parameter								
BW on CL/F	0.56	14.0	-	-	-	-	-	-
ALB on CL/F	-	-	-	-	-	-	1.376	0.410
BW on V/F	0.65	16.5	-	-	-	-	-	-
ALB on V/F	1.33	17.7	-	-	-	-	-	-
BW on CLm/F	0.99	9.3	-	-	-	-	-	-
ALB on CLm/F	-	-	-	-	-	-	1.935	0.514
IIV (SD)	-	-	-	-	-	-	-	-
k_a_	0.89	6.1	-	-	-	-	-	-
CL/F	0.46	3.2	0.40	11.7	0.268	6.3	0.094 ^a^	0.025
V/F	0.52	5.3	0.64	30.4	-	-	-	-
CLm/F	0.52	3.3					0.123 ^a^	0.030
Vm/F	0.62	6.5	-	-	-	-	-	-
Residual error	-	-	-	-	-	-	-	-
Additive error	0.105 (g/L)	16.0	-	-	-	-	34.54	6.040
Proportional error (%)	24.4	2.3	35	4.2	17.5	3.2	31.9	3.4
Modeling software	NONMEM		Monolix Suite		NONMEM		Phoenix	

CL/F, osimertinib apparent oral clearance; V/F, osimertinib apparent volume of distribution; CLm/F, AZ5104 apparent oral clearance; Vm/F, AZ5104 apparent volume of distribution; k_a_, first-order absorption rate constant; ALB, albumin; BW, body weight; IIV, interindividual variability; RSE, relative standard error; SE, standard error; -, not included or not estimated in the source model; ^a^ reported as variance.

### 2.4. Simulation

We conducted simulations based on the final popPK model to evaluate osimertinib exposure across subgroups defined by sex, body weight, and serum albumin.

In this study, we used the minimum steady-state plasma concentration (Cmin,ss) as the exposure metric and pre-specified a literature-informed reference interval: an exploratory pharmacokinetic threshold of 166 ng/mL [24] and a toxicity threshold of 259 ng/mL [14]. These two concentrations serve as reference points for comparison rather than as validated exposure–response boundaries. No established concentration range exists for osimertinib [25]. The exploratory pharmacokinetic threshold of 166 ng/mL was based on the geometric mean reported by the Food and Drug Administration (FDA) [24]. A retrospective cohort study applying this threshold observed no exposure–response relationship [24]. The toxicity threshold of 259 ng/mL was the optimal cutoff of a receiver operating characteristic curve for severe toxicity in a prospective real-world cohort [14].

Simulations based on the finalized model utilized 1000 replicates per simulation scenario to evaluate covariate-specific exposure and accounted for the long half-life of osimertinib, running for a total of 19 days. This duration exceeded the 15 days reported for osimertinib to reach steady state [13]. Steady state was defined as a day-19 trough concentration of at least 99% of the theoretical steady-state trough. Both the approved 80 mg once-daily dose and a reduced 40 mg once-daily dose were simulated.

## 3. Results

### 3.1. Study Population

The included studies consisted of popPK analyses based on the AURA phase I/II and FLAURA clinical trials of adult patients with EGFR-activating mutation NSCLC treated with osimertinib. Using published popPK models and reported summary information from these sources, we generated a virtual cohort that reflected the characteristics of each underlying dataset (Table 1). Baseline characteristics mirrored the source populations, with distributions of key covariates summarized by source. Using data from four published popPK studies, the final model was developed from the MB set; parameter estimates and covariate effects are presented in Table 2.

### 3.2. Final Population PK Model

A one-compartment parent-metabolite PK model with first-order absorption and elimination (ADVAN6 TOL4) was used to describe the PK profile of osimertinib. The population PK model for patients with NSCLC receiving osimertinib was derived using the following equations:

$$\mathrm{CL}/F=\Theta_{\mathrm{CL}}\times {\left(\frac{{\mathrm{BW}}_{i}}{64.57\;}\right)}^{0.105}\times {\left(\frac{{\mathrm{ALB}}_{i}}{3.87\;}\right)}^{0.458}$$

$$V/F=\Theta_{V}\times {\left(\frac{{\mathrm{BW}}_{i}}{64.57}\right)}^{0.249}\times {\left(\frac{{\mathrm{ALB}}_{i}}{3.87\;}\right)}^{0.199}$$

$$\mathrm{CLm}/F=\Theta_{\mathrm{CLm}}\times {\left(\frac{{\mathrm{BW}}_{i}}{64.57\;}\right)}^{0.36}\times {\left(\frac{{\mathrm{ALB}}_{i}}{3.87\;}\right)}^{0.879}\times (1+(-0.217)\times \mathrm{SEX})$$

*Vm*/*F* = *Θ_Vm_* × *e*^(SEX× (−0.457))^

Here, BW*_i_* and ALB*_i_* represent individual body weight and serum albumin, respectively, with covariates centered on median values of 64.57 kg and 3.87 g/dL. SEX was coded as 1 for male and 0 for female. Age, ALT, serum creatinine, and study did not meet the SCM criteria. AST met the SCM criteria but was not retained because its effect was not reproduced in the VAL set and its magnitude was clinically negligible.

### 3.3. Model Validation

We present the final osimertinib PK model parameters with estimates calculated at a 95% confidence interval (CI) through 1000 bootstrap runs. Diagnostics showed a good fit (Table 3). The narrow bootstrap intervals reflect the number of simulated observations rather than the uncertainty of the source estimates.

**Figure 1:**
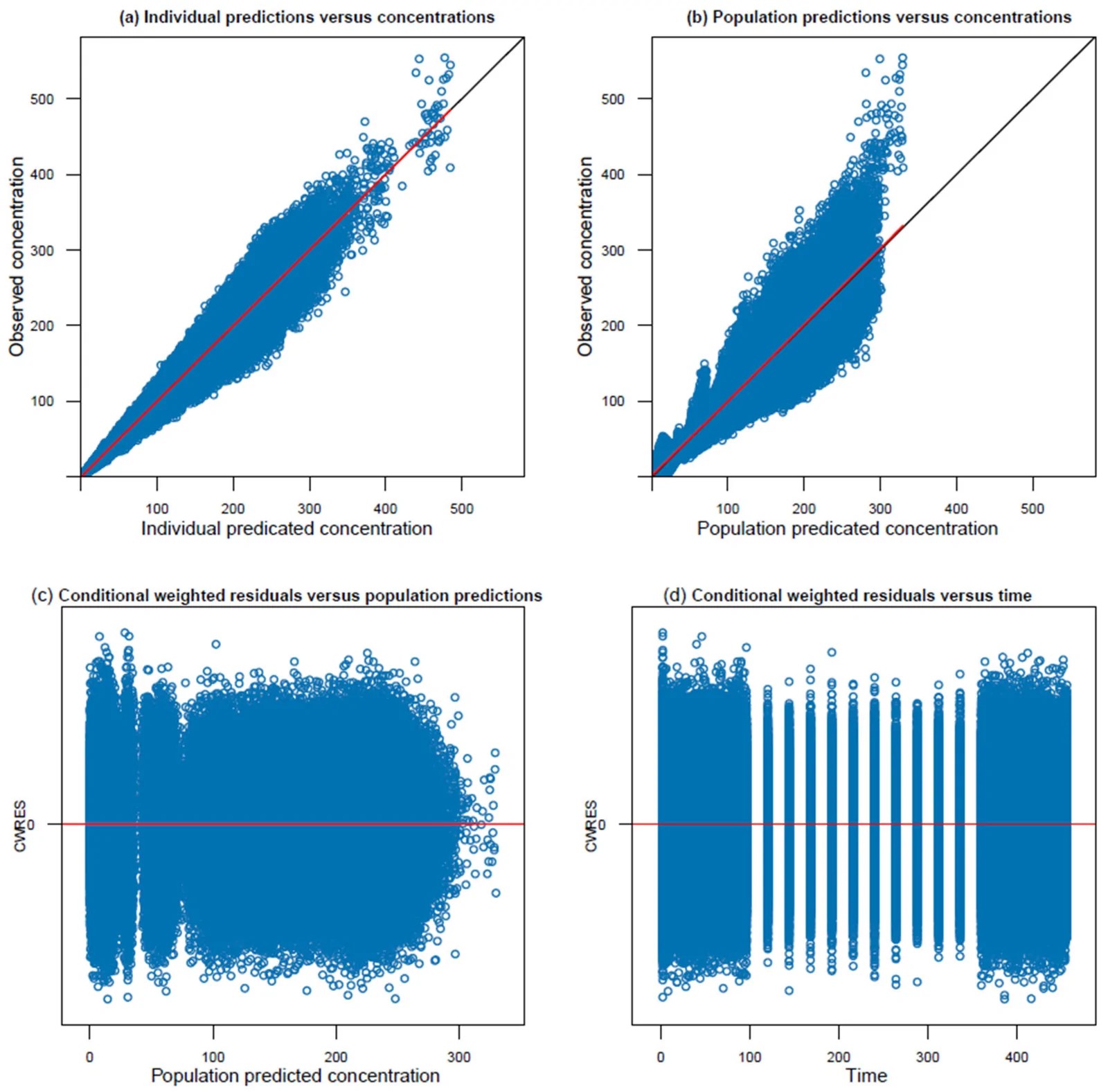
Goodness-of-fit plots for the final population pharmacokinetic model of osimertinib for NSCLC patients. Observed osimertinib concentrations versus (**a**) individual predicted concentration (IPRED) and (**b**) population predicted concentrations (PRED); conditional weighted residuals (CWRES) versus (**c**) population predicted concentrations (PRED) and (**d**) time of osimertinib administration.

Validation indicates a high correlation when predicted values cluster around the identity line with observed values, and the model is considered well-fitted if residuals are within ±3. Goodness-of-fit (GOF) analysis confirmed this high correlation, with residuals within ±3, suggesting a well-fitted model. However, the narrow residual range (±0.3) reflects the simulated data and does not indicate predictive precision in patients (Figure 1).

To assess parameter stability and potential overfitting, we conducted reciprocal cross-validation. The dataset was split into MB (80%) and VAL (20%) subsets, with models fit on each subset to predict the other. For the parent drug, cross-prediction showed RMSE values of 30.16 ng/mL (MB model predicting VAL data) and 32.03 ng/mL (VAL model predicting MB data), with relative RMSE of 16.8% and 17.1%, respectively. For the metabolite, RMSE values were 8.57 ng/mL and 10.68 ng/mL, with relative RMSE of 92.5% and 52.6%. Bias was minimal for the parent drug (within ±5 ng/mL) but showed greater variability for the metabolite (up to −8.56 ng/mL). Prediction of AZ5104 concentrations was less accurate than that of the parent drug. This likely reflects the differing metabolite parameters of the two source models (Table 2). At steady state, AZ5104 circulates at approximately 10% of osimertinib’s plasma exposure [18]. The dose optimization conclusions were based on parent drug Cmin,ss only. Overall, the consistent parent drug RMSE values indicated stable predictive performance across different data subsets (Table S2).

No significant differences were found between the bootstrap-predicted and VPC models for osimertinib pharmacokinetics, both demonstrating reasonable predictive performance. Thus, our model was internally validated using bootstrap and VPC analyses (Table 3 and Figure S1).

### 3.4. Final Model Simulation

The final model was simulated using categorical variables. Body weight was grouped in 10 kg increments from 40 kg to 120 kg. Albumin levels were simulated in 0.5 g/dL increments from 2.5 g/dL to 5.0 g/dL. Both sexes were included, resulting in 108 distinct simulation scenarios. Across the typical parameter values of all 108 scenarios, day-19 troughs exceeded 99% of steady state for both the parent drug and AZ5104.

**Figure 2:**
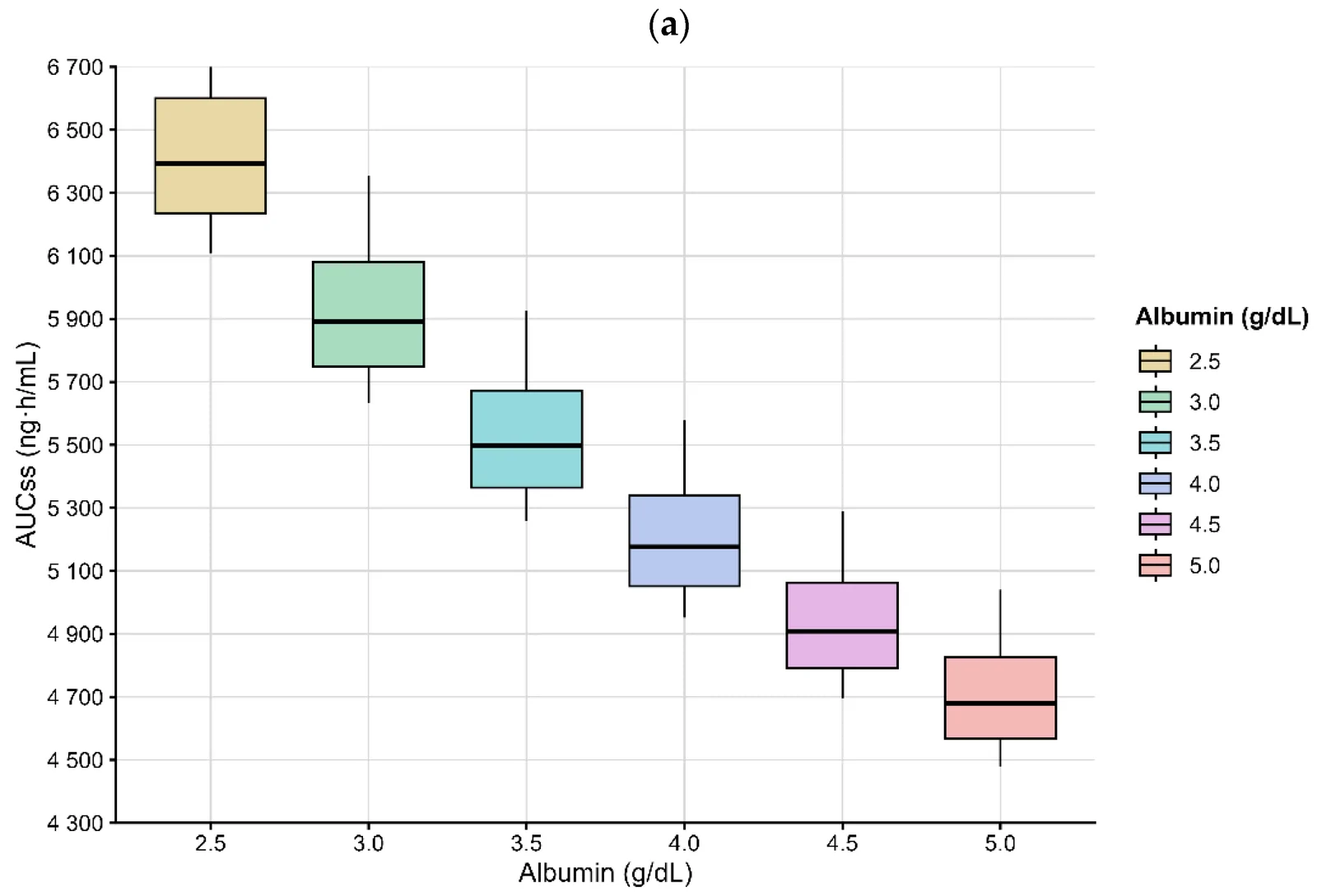

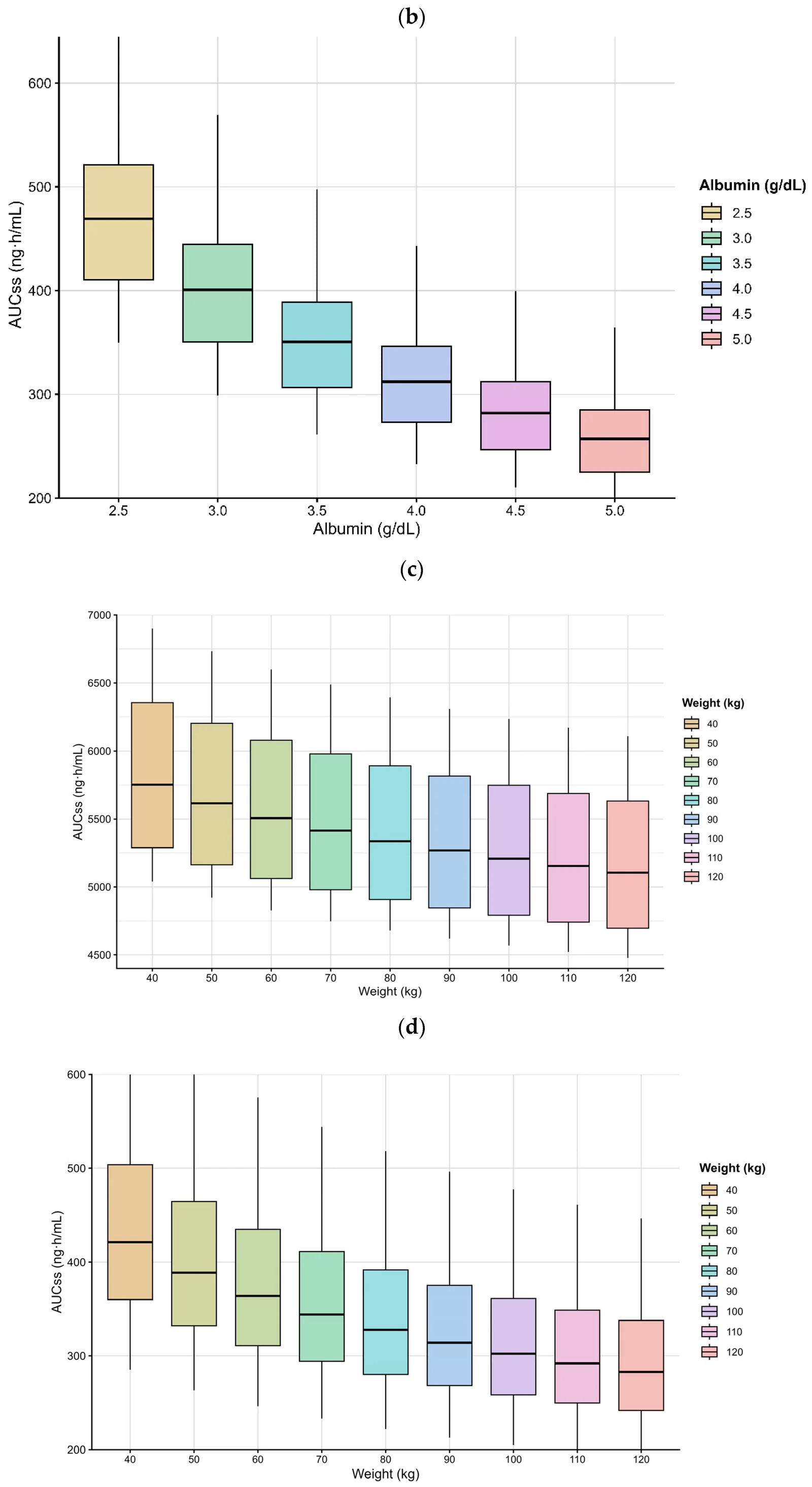
(**a**) Distribution of parent drug AUCss across albumin concentrations; (**b**) distribution of metabolite drug AUCss across albumin concentrations; (**c**) distribution of parent drug AUCss across weight; (**d**) distribution of metabolite drug AUCss across weight. AUC graph by weight for Albumin range (2.5–5.0 g/dL); AUC_ss_, the steady-state area under the curve.

Among individuals with normal albumin levels (4.0 g/dL), steady-state area under the curve (AUC_ss_) values for the parent compound ranged from 4951 to 5579 ng·h/mL, and those for the metabolite ranged from 233 to 443 ng·h/mL. Lower body weight and lower albumin concentrations were associated with higher AUC_ss_ values, while sex had minimal impact on exposure. These values were consistent with those reported in clinical trials of patients receiving 80 mg of osimertinib daily for NSCLC (Figure 2) [26,27].

**Figure 3:**
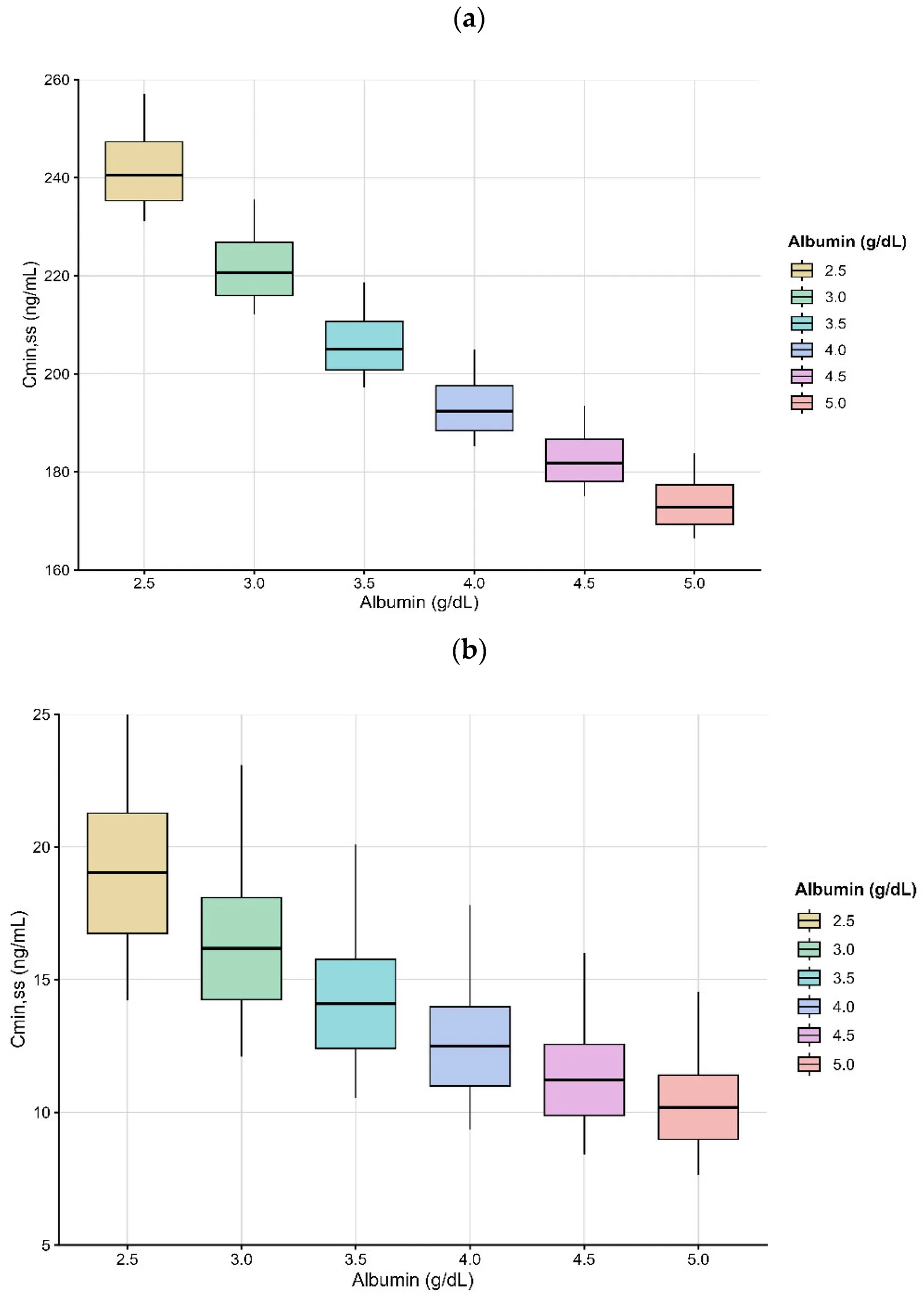
(**a**) Box plot of parent drug Cmin,ss by albumin concentration; (**b**) box plot of metabolite drug Cmin,ss by albumin concentration. Cmin,ss, minimum steady-state plasma concentration.

Due to the long half-life of osimertinib and the need to manage sustained exposure and toxicity risks, we established a reference interval for parent drug Cmin,ss based on literature-informed thresholds: an exploratory pharmacokinetic threshold of 166 ng/mL [24] and a toxicity threshold of 259 ng/mL [14]. Simulation results fell within this reference interval (Figure 3). At 80 mg once daily, none of the 108 scenarios yielded predicted Cmin,ss values below 166 ng/mL or above 259 ng/mL. The highest predicted Cmin,ss was 257.07 ng/mL, observed at a body weight of 40 kg and a serum albumin level of 2.5 g/dL.

## 4. Discussion

This study presents a comprehensive PK analysis of osimertinib in virtual patients with NSCLC, utilizing data from multiple studies to develop an integrated model. Virtual patients were created with specific characteristics typically restricted to real clinical trials. Covariates were selected based on previous studies [9], as well as the FDA [11] and European Medicines Agency (EMA) [28] approval documents, which indicated that demographic factors including body weight and albumin did not require dose adjustment despite their effects on pharmacokinetics.

Our integrated analysis quantified these relationships to characterize exposure variability across patient subgroups. The recommendations in those approval documents rest on the significance of covariate effects within individual analyses. The present simulations instead evaluate whether predicted Cmin,ss remains within the reference interval across the full range of body weight and albumin. This assessment drew on four independently published popPK analyses rather than a single dataset.

The final PK model for osimertinib was a one-compartment parent-metabolite model with first-order absorption and elimination. Key PK parameters were Ka 0.284 h^−1^, CL/F 14.8 L/h, CLm/F 40.1 L/h, V/F 1110 L, and Vm/F 432 L, consistent with values reported in previous studies [8,18]. Body weight and serum albumin were significant covariates influencing osimertinib PK, likely due to their effects on plasma protein binding.

Although body weight and albumin were statistically significant covariates, their clinical impact on osimertinib exposure was limited. Monte Carlo simulations across diverse patient characteristics suggested that variations in Cmin,ss related to weight, albumin, and sex remained within the reference interval at the standard 80 mg dose [8,18,29].

Osimertinib exhibits high plasma protein binding (approximately 95%), although the clinical implications of this property for exposure variability require further investigation. While Cmin,ss below 259 ng/mL is relevant to minimizing toxicity [14], our simulation results suggest that the standard 80 mg dose maintains predicted Cmin,ss within the literature-informed reference interval across the evaluated body weight and albumin scenarios, supporting current dosing recommendations rather than providing a direct clinical recommendation for dose modification. Although prior studies have shown that dose reduction to 40 mg in patients with concentrations exceeding 259 ng/mL can decrease severe toxicity risk by 53% [14], the highest predicted Cmin,ss at 80 mg was 257.07 ng/mL, approaching this threshold at the lowest body weight and albumin combination. This finding highlights a potential exposure-related safety concern under the combined conditions of severe hypoalbuminemia and low body weight, although its clinical implications require prospective validation. In the same combination, simulation of 40 mg once daily predicted a Cmin,ss of 129.1 ng/mL, below both the toxicity threshold of 259 ng/mL and the exploratory pharmacokinetic threshold of 166 ng/mL [24]. The model predictions suggest that Cmin,ss remained within the reference interval with standard dosing across all 108 simulated covariate scenarios. However, monitoring may still be valuable in select clinical situations, such as suspected non-adherence, drug–drug interactions, or unexplained toxicity.

The PK parameter estimates from our integrated model are broadly consistent with those reported in a recent comprehensive popPK analysis by Johnson et al. [30], who analyzed 1689 patients across the AURA, FLAURA, and ADAURA clinical programs. Their median Cmin,ss values of approximately 175–218 ng/mL across adjuvant to later-line settings fall within the reference interval of 166–259 ng/mL. This consistency supports the plausibility of our integrated virtual-patient approach, despite the absence of direct patient-level data. Two independent real-world cohorts not used for model development reported median osimertinib trough concentrations of 211 and 227 ng/mL, respectively, both within the literature-informed reference interval [24,26].

Collectively, these findings highlight the potential of virtual patient modeling in addressing clinical research limitations, such as participant shortages and diverse patient conditions. The model developed here provides a basis for evaluating osimertinib dosing in patients with NSCLC and can guide future research. Despite its strengths, this study has several limitations. Reliance on published population pharmacokinetic models and reported summary information rather than individual patient-level data may limit the ability to fully capture interindividual variability. The uncertainty of the source estimates was not carried into the final estimates or the predicted exposures. Additionally, integrating pharmacokinetic parameters from studies with different reporting formats required standardization that may introduce uncertainty. Rigorous standards for data conversion and virtual patient generation are essential to ensure accuracy [19]. Body weight for the cohort of Rodier et al. [20] was derived from BMI and did not affect the concentrations simulated from its covariate-free model, but these body weight values were included in the integrated dataset used to estimate the body weight effect.

The four source analyses did not agree on whether body weight and serum albumin affected osimertinib pharmacokinetics. Two analyses retained at least one of these covariates, and two retained neither. The body weight and albumin effects reported here therefore reflect all four source models rather than a single covariate model. Covariates were sampled from the marginal distributions reported in each source publication, since neither joint distributions nor correlation matrices were available. Physiological correlations between body weight and serum albumin were therefore not reproduced, and uncommon covariate combinations may have been generated. Exposure was nevertheless evaluated across a full factorial grid that included the lowest body weight and albumin values, at which predicted exposure was highest.

While cross-validation demonstrated stable predictive performance for the parent drug, our findings were derived from virtual patients reconstructed from clinical trial populations, which may not fully represent real-world patient diversity. Because both subsets derive from the same underlying model parameters, this cross-validation constitutes internal validation of parameter stability rather than external validation. Although the consistency of our PK estimates with the aforementioned real-world and clinical trial cohorts provides indirect support for generalizability, formal external validation using independent patient-level data was not performed. Moreover, the integrated analysis included only four source popPK studies, which may introduce selection bias toward populations meeting specific clinical trial eligibility criteria. Both reference concentrations (166 and 259 ng/mL) were derived from observational cohorts, and their applicability across treatment settings and patient populations has not been established.

Concomitant medications and the treatment line were not reported in all four source studies and could not be evaluated. Furthermore, our model focused on demographic and physiological covariates but did not incorporate genetic factors such as polymorphisms in drug-metabolizing enzymes or transporters that may influence osimertinib pharmacokinetics. Although Rodier et al. [20] found no significant association between CYP3A4/5 or transporter polymorphisms and osimertinib clearance in a predominantly Caucasian cohort, pharmacogenomic variability may be more clinically relevant in East Asian populations, where the allele frequencies of CYP3A4 and ABCG2 variants differ substantially [21,31]. Future research should conduct prospective external validation with real-world patient-level data across diverse ethnic populations and incorporate pharmacogenomic covariates to assess whether genotype-guided dose individualization improves clinical outcomes in specific subgroups.

## 5. Conclusions

This is the first integrated population pharmacokinetic analysis to develop a comprehensive model for osimertinib in virtual patients with NSCLC. These findings should be interpreted as model-informed and hypothesis-generating rather than clinically validated evidence. The simulations suggest that the standard 80 mg dose maintains predicted Cmin,ss within the reference interval across the simulated range of body weight and albumin, supporting current dosing recommendations without routine dose adjustment. Beyond this confirmation, this analysis quantifies how closely predicted exposure approaches the limits of the reference interval, with the highest predicted Cmin,ss of 257.07 ng/mL occurring at a body weight of 40 kg and a serum albumin level of 2.5 g/dL, close to the literature-informed toxicity threshold of 259 ng/mL. Prospective validation in real-world clinical settings is warranted to confirm generalizability.

The developed model provides a framework for understanding osimertinib pharmacokinetics and can inform prospective evaluation of dose management in patients with NSCLC. This integrated modeling approach demonstrates the utility of integrating data from multiple studies to evaluate drug exposure across heterogeneous populations when individual patient-level data is unavailable, consistent with prior applications of this methodology to other agents, including erlotinib in NSCLC [15,16,17].

## Figures and Tables

**Table 3:** Parameter estimations and bootstrap results of the final osimertinib model.

Parameter	Estimate	%RSE	Bootstrap Median	95% CI Bootstrap	
				Lower	Upper
k_a_ (h^−1^)	0.284 FIX	-	0.284 FIX	-	-
CL/F (L/h)	14.8	0.012	14.8	14.7112	14.8303
V/F (L)	1110	0.02	1105.7	1100.444	1111.22
CLm/F(L/h)	40.1	0.34	40.0	38.2810	41.7978
Vm/F (L)	432	0.74	432.0	405.7202	459.8526
Covariate effect on parameter					
BW on CL/F	0.105	11	0.1045	0.08367	0.1262
BW on V/F	0.249	7	0.2491	0.2163	0.2841
BW on CLm/F	0.36	22	0.3590	0.20835	0.51596
ALB on CL/F	0.458	5	0.4569	0.41302	0.50079
ALB on V/F	0.199	12	0.1996	0.1536	0.24811
ALB on CLm/F	0.879	11	0.8788	0.6761	1.0416
SEX on CLm/F	−0.217	11	−0.2163	−0.25927	−0.16861
SEX on Vm/F	−0.457	9	−0.4566	−0.54015	−0.37359
IIV					
CL/F	0.0124	3	0.01234	0.011608	0.01310
V/F	0.0208	3	0.02078	0.019501	0.02214
CLm/F	0.346	2	0.3456	0.33344	0.35778
Vm/F	0.763	1	0.7617	0.740718	0.78069
Residual error					
Additive error	7.99 × 10^−8^ FIX	-	7.99 × 10^−8^ FIX	-	-
Proportional error	0.0912	0.22	0.09122	0.090996	0.09144

Successful runs from 1000 bootstrap resamplings of osimertinib data.

## Data Availability

The code and simulated datasets used in this study are openly available on GitHub at https://github.com/IM-JW/Osimertinib_integrated_PK (accessed on 17 October 2026). The repository includes the mrgsolve implementations of the four source models, NONMEM control files, simulated analysis datasets, and R scripts for data processing, model evaluation, simulation, and figure generation.

## Supplementary Materials

The following supporting information can be downloaded at https://www.mdpi.com/article/10.3390/pharmaceutics18101332/s1, Table S1: PICO method for searching literature; Table S2: Cross-validation results for population pharmacokinetic model performance; Figure S1: Visual predictive checks (VPCs) of osimertinib’s final population pharmacokinetic model for NSCLC patients.
