# Supplementary material for: Osimertinib Optimal Dosing Through Population Pharmacokinetics in Virtual Patients with NSCLC: An Approach Integrating Multiple Studies
Source: Pharmaceutics. 2026 Oct 20;18(10):1332. doi: 10.3390/pharmaceutics18101332 (PMC13708703; doi:10.3390/pharmaceutics18101332)
Supplement: Supplementary file 1 [file pharmaceutics-18-01332-s001.zip › pharmaceutics-4560344-supplementary.pdf]

Supplementary Materials: Osimertinib Optimal Dosing through Population Pharmacokinetics in Virtual Patients with NSCLC: an Approach Integrating Multiple Studies

Ji Woo Lim [1,2], Heungjo Kim [2], Hongjae Lee [2,3], Hyeeun Kim [2,4], Kyubin Kim [2], Kyongkuk Ryu [2] and Min Jung Chang [1,2,3,4]*

Table S1. PICO method for searching literature.

Table S2. Cross-Validation Results for Population Pharmacokinetic Model Performance.

Figure S1. Visual predictive checks (VPCs) of osimertinib's final population pharmacokinetic model for NSCLC patients.

Table S1. PICO method for searching literature.

**Pubmed**	
PICO method	Key Word
P (Participants)	("Carcinoma, Non-Small-Cell Lung"[Mesh]) OR "Lung Neoplasms"[Mesh] ((lung neoplasms[MeSH Terms]) OR (lung cancer[Title/Abstract]) OR (lung carcinoma[Title/Abstract]) OR (NSCLC[Title/Abstract]) OR (non-small cell lung cancer[Title/Abstract]) OR (lung tumor[Title/Abstract]) OR (lung tumour[Title/Abstract]) OR (pulmonary neoplasm[Title/Abstract]) OR (pulmonary carcinoma[Title/Abstract]) OR (pulmonary tumor[Title/Abstract]) OR (pulmonary tumours[Title/Abstract]))
I (Intervention)	((osimertinib[Title/Abstract]) OR (tagrisso[Title/Abstract])) OR (L01EB04[Title/Abstract])
C (Comparison)	
O (Outcome)	(pharmacokinetic[Title/Abstract]) OR (pharmacokinetics[Title/Abstract])) OR (population pharmacokinetic[Title/Abstract])) OR (population pharmacokinetics[Title/Abstract])) OR (PK[Title/Abstract])) OR (pop PK[Title/Abstract])) OR (dose finding study[Title/Abstract])) OR (dose finding[Title/Abstract])) OR (dose optimization[Title/Abstract])) OR (exposure-response[Title/Abstract])) OR (exposure-response analysis[Title/Abstract])

**Embase**	
PICO method	Key Word
P (Participants)	'non small cell lung cancer'/exp OR 'non small cell lung cancer' OR (non AND small AND ('cell'/exp OR cell) AND ('lung'/exp OR lung) AND ('cancer'/exp OR cancer))

https://doi.org/10.3390/pharmaceutics18101332

I (Intervention)	'osimertinib'/exp OR osimertinib OR 'tagrisso'/exp OR tagrisso
C (Comparison)	
O (Outcome)	'pharmacokinetics'/exp OR pharmacokinetics OR 'dose optimization'/exp OR 'dose optimization' OR 'exposure respons' OR 'dose calculation'/exp OR 'dose calculation'

**Cochrane**	
PICO method	Key Word
P (Participants)	(non smell cell lung cancer):ti,ab,kw OR (NSCLC):ti,ab,kw OR (cancer):ti,ab,kw
I (Intervention)	(osimertinib):ti,ab,kw OR (tagrisso):ti,ab,kw
C (Comparison)	
O (Outcome)	("pharmacokinetics"):ti,ab,kw OR ("dose optimization"):ti,ab,kw OR ("dose calculation"):ti,ab,kw AND ("exposure response" OR "exposure–response"):ti,ab,kw
	* ti,title; ab,abstract; kw,keyword

https://doi.org/10.3390/pharmaceutics18101332

Table S2. Cross-validation results for population pharmacokinetic model performance.

Model/Dataset	RMSE (ng/mL)	MAE (ng/mL)	Bias (ng/mL)	rRMSE (%)	rMAE (%)	rBias (%)
Parent Concentration						
Test model/Validation data	30.16	21.90	-1.39	16.8	12.2	-0.8
Validation model/Test data	32.03	24.22	-4.82	17.1	12.9	-2.6
Metabolite Concentration						
Test model/Validation data	8.57	6.83	-1.44	92.5	73.8	-15.6
Validation model/Test data	10.68	8.66	-8.56	52.6	42.7	-42.2

RMSE, root mean square error; MAE, mean absolute error; rRMSE, relative RMSE; rMAE, relative MAE; rBias, relative bias.

https://doi.org/10.3390/pharmaceutics18101332

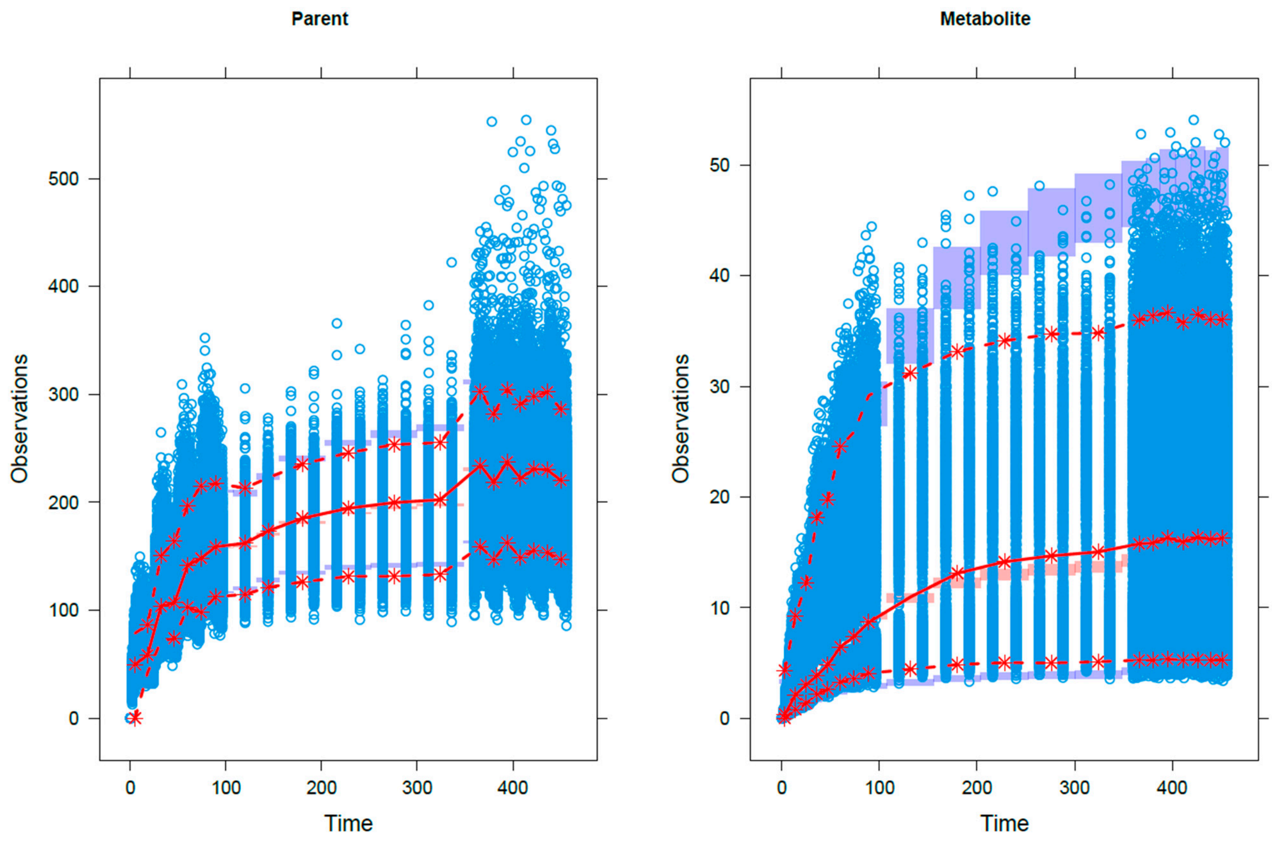

Figure S1. Visual predictive checks (VPCs) of osimertinib's final population pharmacokinetic model for NSCLC patients. Observed osimertinib concentrations; solid line, median; lower and upper dashed lines, 5th and 95th percentiles of the observed data, respectively; shaded areas, 90% confidence intervals for simulated predicted median, 5th, and 95th percentiles constructed from 1,000 simulated datasets of individuals from the original data.

https://doi.org/10.3390/pharmaceutics18101332
